# Microleakage in conservative cavities varying the preparation method and surface treatment

**DOI:** 10.1590/S1678-77572010000400017

**Published:** 2010

**Authors:** Juliana Abdallah ATOUI, Michelle Alexandra CHINELATTI, Regina Guenka PALMA-DIBB, Silmara Aparecida Milori CORONA

**Affiliations:** 1 DDS, MSc, Department of Restorative Dentistry, Ribeirão Preto Dental School, University of São Paulo, Ribeirão Preto, SP, Brazil.; 2 DDS, MSc, PhD, Department of Restorative Dentistry, Ribeirão Preto Dental School, University of São Paulo, Ribeirão Preto, SP, Brazil.; 3 DDS, MS, PhD, Associate Professor, Department of Restorative Dentistry, Ribeirão Preto Dental School, University of São Paulo, Ribeirão Preto, SP, Brazil.

**Keywords:** Dental air abrasion, Dental cavity preparation, Marginal adaptation, Dental etching

## Abstract

**Objective:**

To assess microleakage in conservative class V cavities prepared with aluminum-oxide air abrasion or turbine and restored with self-etching or etch-and-rinse adhesive systems.

**Material and Methods:**

Forty premolars were randomly assigned to 4 groups (I and II: air abrasion; III and IV: turbine) and class V cavities were prepared on the buccal surfaces. Conditioning approaches were: groups I/III - 37% phosphoric acid; groups II/IV -self-priming etchant (Tyrian-SPe). Cavities were restored with One Step Plus/Filtek Z250. After finishing, specimens were thermocycled, immersed in 50% silver nitrate, and serially sectioned. Microleakage at the occlusal and cervical interfaces was measured in mm and calculated by a software. Data were subjected to ANOVA and Tukey’s test (α=0.05).

**Results:**

Forty premolars were randomly assigned to 4 groups (I and II: air abrasion; III and IV: turbine) and class V cavities were prepared on the buccal surfaces. Conditioning approaches were: groups I/III - 37% phosphoric acid; groups II/IV -self-priming etchant (Tyrian-SPe). Cavities were restored with One Step Plus/Filtek Z250. After finishing, specimens were thermocycled, immersed in 50% silver nitrate, and serially sectioned. Microleakage at the occlusal and cervical interfaces was measured in mm and calculated by a software. Data were subjected to ANOVA and Tukey’s test (α=0.05).

**Conclusion:**

Marginal seal of cavities prepared with aluminum-oxide air abrasion was different from that of conventionally prepared cavities, and the etch-and-rinse system promoted higher marginal seal at both enamel and dentin margins.

## INTRODUCTION

The concept of cavity preparation has been modified and/or replaced by more conservative preparations due to the use of new techniques and restorative materials that preserve sound tooth structure by minimizing the necessity to enlarge the cavity preparation^11^. Aluminum oxide air abrasion is one of the methods used for the preparation of conservative cavity designs. Air-abrasive technique using a high-speed stream of purified aluminum oxide particles delivered by air pressure for removal of tooth structure was reintroducedwith the intention to eliminate pressure, heat, noise and vibration associated with rotary instruments, and to reduce pain, allowing preparation with less need for local anesthesia^20^.

Contemporary restorative techniques are based on the adhesive properties of resin-based materials. The basic mechanism of bonding to enamel and dentin is an exchange process involving replacement of minerals removed from the hard tissue by resin monomers that upon setting become micro-mechanically interlocked in the created porosities^23^.

Following an etch-and-rinse approach, the tooth is first etched and rinsed off. This conditioning step treats enamel and dentin with inorganic acid (mostly 30-40% phosphoric acid), promoting the increase of the permeability and the demineralization of enamel as well as dentin (inter-, peritubular, intratubular)^23^. However, these systems produce a deep demineralized area, without the certainty of complete infiltration of monomers into the exposed collagen fiber mesh, thus affecting the longevity of adhesion^12^.

Self-etching adhesives use non-rinse acidic monomers that simultaneously condition and prime dentin, eliminating the acid-conditioning step and reducing the technique sensitivity^19^. This technique avoids the formation of extensive demineralized areas, which may not be fully impregnated by monomers^24^. According to the literature, the incorporation of smear layer to the adhesive interface can result in a more defective adhesion area^12^.

The most cited reasons for failure of adhesive restorations placed with earlier adhesives are loss of retention and marginal adaptation^7^. The behavior of restorative materials in cavities prepared with aluminum oxide air abrasion has been extensively investigated^4,6-8,21,25^, and, in general, differences have not been observed between the restorations of cavities prepared with air abrasion or turbine followed by acid conditioning^6,25^. The restorations that did not receive acid conditioning after preparation with air abrasion presented increase in microleakage^4,7,8^. In addition, better marginal seal has also been observed in enamel margins of cavities prepared with air abrasion^4,10,21^.

Despite the advances in restorative dentistry, there is only a limited number of studies^8^ reporting the effectiveness of self-etching adhesive systems in the sealing ability of cavities prepared with air abrasion. Thus, the present study evaluated *in vitro* the degree of microleakage in conservative cavities prepared with air abrasion and restored using a self etching adhesive system. The hypothesis of this study was that conservative class V cavities prepared with air abrasion and restored with a self-etching primer have less marginal microleakage.

## MATERIAL AND METHODS

The research protocol was approved by the Research ethics Committee of Ribeirão Preto Dental School, University of São Paulo (process # 2003.1.312.58.9).

Forty sound human premolars, extracted within a 6-month period and stored in 0.9% saline solution at 4°C, were examined to confirm the absence of defects in enamel and dentin, and selected for the study. Teeth were carefully cleaned with a hand scaler and water-pumice slurry in prophylaxis rubber cups, and randomly assigned to 4 groups (n=10), according to the cavity preparation method and conditioning approach. The experimental groups were as follows: Group I: Air abrasion + Phosphoric acid; Group II: Air abrasion + Self-priming etchant; Group III: Turbine + Phosphoric acid; Group IV: Turbine + Self-priming etchant.

Forty non-carious class V cavities were prepared on buccal surfaces with the occlusal margin located in enamel and cervical margin in dentin/cementum. The cavity outline was previously traced on surfaces with a OHP marker pen, to define a uniform size (3 mm mesiodistal width and 1.5 mm occlusogingival dimensions). The depth of the cavity was approximately 1.5 mm, as measured with a premarked periodontal probe.

Following the pre-defined dimensions The airabraded cavities were prepared with the air-abrasive system Mach 4.1 (Kreativ Inc., Albany, OR, USA) with aluminum oxide particles of 27.5 mm under 60 psi pressure with an intensity of 4 g/min at continuous mode, delivered by a 0.011-inch nozzle opening, with a 90° angle with the tooth surface. The application distance was standardized using a custom designed apparatus consisting of a moving holder that positioned the handpiece in such a way that the aluminum oxide particle stream was delivered at a constant distance of 2 mm from the delimited surface of the specimen. The specimens were fixed with wax at a semi-adjustable base. The operator manipulated the apparatus' screws in such way that the semiadjustable base with the specimen was moved in right-to-left and forward-to-back directions, thereby allowing the stream to provide a more accurate application of the entire delimited site. After the stream application, the specimen was removed and the cavity was rinsed for 30 s, and then gently dried with oil-free compressed air.

For cavities prepared with turbine, a #330 carbide bur was used (JeT Brand; Beavers Dental, Morrisburg, Canada) at a high-speed handpiece (Silent MRS; Dabi Atlante S.A. Ind. Med. Odontológica, Ribeirão Preto, SP, Brazil) under water spray. Cavity finishing was done with the same bur at a low-speed handpiece. The enamel cavosurface angle was beveled with a #1195-diamond bur.

After cavity preparation, the enamel/dentin surfaces were conditioned according to the experimental group. Specifications of the materials employed for surface treatment are shown in Figure 1. For groups I and III, the cavities were etched with 37% phosphoric acid gel (Figure 1) for 30 s for enamel and 15 s for dentin, washed with water spray for 30 s and dried with absorbing paper, providing a moist surface. Groups II and IV were conditioned with the self-priming etchant Tyrian SPe (Figure 1). equal amounts of part A and B were mixed thereby generating a purple color. Then, the mixture was applied with a foam pellet for 10 s, and the excess was removed until the purple color had completely disappeared.

**Figure 1:** Specifications of the materials and equipments employed

**Material**	**Composition**	**Batch no.**	**Manufacturer**
			
Etch 37	37% phosphoric acid	E- 5503EBM	Bisco Inc., Schaumburg, IL, USA
			
Tyrian SPE	Part A: 2-Acrylamido, 2-methyl propanesuflonic acid, Bis-2-methacryloyloxyethyl phosphate. Part B: ethanol	SPEA 030000056 SPE B 0300002288	Bisco Inc., Schaumburg, IL, USA
			
Filtek™ Z250	BIS-GMA, UMTA, BIS-EMA		3M ESPE, St. Paul, MN, USA

Following this, One-Step Plus adhesive system (Figure 1) was applied by two consecutive coats, brushed for 10 s, gently blot-dried for 10 s to evaporate the solvent, and light-cured for 10 s with a visible light-curing unit with a 450 mW/cm^2^ output (XL 3000, 3M eSPe, St Paul, MN, USA). The cavities were restored with a hybrid light-activated composite resin (Z250, 3M; shade A3.5), inserted in three increments (maximum thickness of 2 mm). The first two increments were applied obliquely against the occlusal and the gingival walls, respectively. The final increment was inserted following tooth contouring. each increment was light-cured for 40 s with the same visible light-curing unit.

Specimens were stored for 24 h in distilled water at 37°C and then the restorations were polished with Super-Snap disks (Shofu Inc., Kyoto, Japan) in a decreasing abrasive order. All cavity preparations, restorations and finishing procedures were performed by the same operator.

The specimens were subjected to a thermocycling regimen of 500 cycles between 5°C and 55°C waterbaths. Dwell time was 1 min, with a 3-s transfer time between baths. In preparation for the dye penetration test, the teeth were dried superficially, had the apices sealed off with epoxy resin and the entire tooth received two coats of nail varnish, except for a 2-mm window around restoration margins. As the nail varnish dried, the teeth were immersed in distilled water for 2 h, and then immersed in a 50% aqueous silver nitrate solution for 8 h, kept in a light-proof container. Next, the teeth were rinsed thoroughly in tap water and the nail varnish was entirely removed with a sharp instrument.

The specimens were embedded in chemically activated acrylic resin (JeT, Clássico, São Paulo, SP, Brazil) and sectioned longitudinally in a mesiodistal direction with a water-cooled diamond saw, in a sectioning machine (Minitom; Struers A/S, Copenhagen, Denmark). Then, they were embedded again in acrylic resin blocks and sectioned in a buccolingual direction, providing two to three 1.0-mm-thick sections for each tooth. Then, the sections were exposed to the light of a photoflood lamp for 20 min (115 V, 500 W) to reveal the silver nitrate, which, exposed to light, acquires a dark color, allowing the visualization of the tracer-penetrated areas. The sections were initially thinned in a polishing machine (Struers A/S) with 180- to 600-grit silicon carbide paper, and then manually smoothed with 1000- to 1200-grit SiC paper to obtain a flat surface and a final thickness of approximately 0.25 mm.

The cuts were identified and carefully fixed on microscopic slides, and the margins were analyzed separately; each margin was viewed under a x5 magnification optical microscope (Axiostar Plus, Carl Zeiss Vision GmbH, München-Hallbergmoos, Germany) connected to a digital camera (Cybershot 3.3 MPeG Movie eX, model no. DSC-S75, Sony Corporation, Tokyo, Japan). The images obtained were transmitted to a personal computer and, after digitization, they were analyzed by Axion Vision 3.1 software (Carl Zeiss Vision GmbH), which performs a standardized assessment of the tracer’s extent along the tooth-composite-interface and allows a quantitative measurement in millimeters. The dye penetration depth along the cavity wall (including both occlusal and cervical margins) of each cut was measured. Microleakage at each interface was obtained by calculating the ratio (percent value) of the tracer penetration along the tooth-restoration interface and the total length of the enamel and/ or dentin interface. Tracer penetration at enamel and dentin interfaces was calculated separately for each section, and then the mean for each tooth was determined.

Data were analyzed statistically by three-way ANOVA followed by Tukey’s test for pair-wise multiple comparisons at a 0.05 significance level.

## RESULTS

The means of dye penetration and standard deviations at both interfaces for each experimental group are shown in Table 1.

**Table 1:** Mean values and standard deviations of microleakage for enamel- and dentin/cementum-composite-interface according to the preparation method and surface treatment

**Preparation method**	**Margins**	**Phosphoric acid**	**Self-priming etchant**
			
Air abrasion	Enamel	6.07 (7.57) a A	34.45 (15.36) b A
Dentin/cementum	31.70 (15.52) aC	54.54 (30.06) b A	
Turbine	Enamel	15.04 (9.33) a AB	32.91 (9.90) b A
Dentin/cementum	18.53 (12.32) a BC	42.75 (21.10) b A	

Three-way ANOVA revealed statistically significant differences (p<0.05) for the margin types and conditioning approaches (p<0.05), and that the margin in enamel and total-etching system had significantly lower microleakage.

Analyzing the interaction of factors, the etch and-rinse system showed statistically significant difference (p<0.05) between enamel and dentin/ cement margins in the air-abraded cavities, but not in the bur-prepared cavities. Groups II (air abrasion + Tyrian SPe) and IV (turbine + Tyrian SPe) showed higher microleakage degrees on both margins and different significantly from groups I and III.

## DISCUSSION

The findings of this work showed that air abrasion technique for cavity preparations provided different seal at the margins of composite resin restorations compared the turbine. This fact may be due to the macro and microscopic irregularities in the abraded surface, which may have influenced in the mechanical interlocking. Moreover, the roughened surface resulting from air abrasion preparation limits the penetration of the adhesive agent when this modified surface is not etched with acid^9^. In addition to these factors, the permanence of aluminum oxide particles on the abraded surface can also influence the penetration of the adhesive. A scanning electronic microscopy study^22^ revealed that the morphology of the adhesive interfaces of the lateral walls of airabraded cavities was similar to the obtained with bur-prepared. However, the adhesive interfaces of the pulpal walls were more irregular in the air abrasion preparations. Several studies^1,4,8,21,25^ have found a decrease in marginal seal in cavities prepared with air abrasion, mainly when 27-µm-diameter particles were used^6^. Another important aspect to be observed in the present study is that the absence of finishing in cavities prepared with air abrasion probably no interferes in the adaptation of the restorative material. Corona, et al.^4^ (2001) verified that air abrasion preparation do not promote a well define contour in cavity margins and walls that can adversely influence in the marginal seal of the restorations.

In the present work, differences were observed between enamel and dentin/cementum margins, only for phosphoric acid. This could be ascribed to morphological and structural differences of the substrates, as well as their distinct composition. Such results corroborate the findings of the literature^4,16,26^ which report better behavior of enamel surface as for adhesive resistance as for marginal microleakage.

Another important aspect that must be taken into account is the polymerization shrinkage of the resinous restorative material. Despite conservative preparations and the incremental composite insertion technique, as performed in this study, thereby reducing the amount of material and the polymerization shrinkage, it was not possible the completely seal both cavity margins. It is known that the resultant stress of polymerization shrinkage of composite resin can generate tensions between the restorative material and tooth substrate, which, consequently, can generate gaps in the adhesive interface. This stress depends on some factors, such as the cavity configuration (factor C), and can reach around 10 to 15 MPa^5^. In spite of the care with regard to this factor, Class V restorations present a relatively small factor C that results in less stress in the adhesive interface, creating less gaps and subsequent less microleakage^14^.

In the overall and independent analysis of the studied factors, the surface treatment with the etchand-rinse system provided better marginal seal than the self-etching system. This fact may be explained by the surface morphological pattern created after phosphoric acid application. At enamel, this acid promotes a demineralization, which produces an intra- and interprismatic dissolution that results in irregularities, through which the adhesive agent can flow and form a micromechanical interaction due to the formation of resinous tags after its polymerization^3^. At dentin, this acid completely removes the smear layer, demineralizing the peri- and intertubular dentin, widening dentinal tubule openings and increasing the permeability of this substrate^17^.

According to Hannig, et al.^9^ (2004), tooth surface treatment with self-etching agents does not remove the smear layer significantly, since it is not carried through the washing of the surface, remaining some regions without treatment^13^. In addition, selfetching systems consist of weak acids The reactive components in the primers of these systems consist of phosphates derived from hydrophilic monomers^10^ that are able to treat and penetrate simultaneously in the enamel surface, but not in a homogeneous way^9^.

In this study, for both preparation methods, enamel margins showed higher microleakage values when the self-etching system was applied. A feasible explanation for such behavior is the lower capacity of these systems to etch as 35% or 37% phosphoric acid, due to the relatively higher pH of these selfetching agents (1.5 - 3.0) as compared to phosphoric acid (pH 0.02 – 0.42)^18^. In dentin, it is known that a conditioning agent with low pH, as phosphoric acid, provides more demineralization^1^.

At dentin/cementum margins, this study disclosed that the conventional preparation with turbine, associate to the etch-and-rinse system, still could not be replaced by air abrasion preparation. The particle stream acts indiscriminately in the organic and inorganic portions of the substrate, leading to surface roughness and tubule obliteration, and consequently absence of resinous tags, compromising the adhesive layer and the marginal seal^2,15^.

The findings of this study revealed a distinct behavior of the adhesive systems, and showed that the aluminum oxide air abrasion did not influence significantly the marginal seal of conservative class V restorations, with improved results in enamel after acid etching. It is important to emphasize that, clinically, no matter its length, the presence of significant leakage is the problem. Also, the intrinsic aspects related to adhesive systems, such as the pretreatment technique required, composition and mechanism of adhesion, may decisively influence their effectiveness in sealing cavity margins and preventing marginal microleakage.

Comparison with the literature is difficult due to the lack of studies assessing the factors studied in this work. Thus, further studies are necessary to investigate the properties of restorative materials as well as other aspects of cavity preparation and tooth conditioning.

## CONCLUSIONS

Based on the results obtained in this study, it may be concluded that: conditioning of enamel and dentin with phosphoric acid provided better marginal seal than the self-etching approach; marginal seal of class V cavities prepared with air abrasion was different from that of conventional turbine preparation; there was higher marginal seal of enamel margins than dentin/cementum margins for groups I and III.
